# Association between the Fibrosis-4 index and mortality risk in acute pancreatitis

**DOI:** 10.3389/fmed.2026.1767483

**Published:** 2026-05-08

**Authors:** Lijie Wang, Ruoran Wang, Yan Kang, Xiaofeng Ou

**Affiliations:** 1 Department of Critical Care Medicine, West China Hospital, Sichuan University, Chengdu, Sichuan, China; 2 Department of Neurosurgery, The First Medical Centre, Chinese PLA General Hospital, Beijing, China

**Keywords:** acute pancreatitis, Fibrosis-4, logistic regression, mortality, predictive model

## Abstract

**Background:**

This study aimed to evaluate the predictive value of the Fibrosis-4 (FIB-4) index for 28-day mortality in patients with acute pancreatitis and to develop a comprehensive prognostic model.

**Methods:**

This retrospective study included 467 adult patients admitted to the intensive care unit. Risk factors for 28-day mortality were identified through univariate and subsequent multivariate logistic regression analyses. Based on the significant independent predictors, a predictive logistic regression model was formulated.

**Results:**

Multivariate analysis revealed that age, APACHE II score, WBC, serum sodium, and the FIB-4 index were independent predictors of 28-day mortality. The combined model incorporating these five factors achieved an area under the receiver operating characteristic curve (AUC) of 0.80, which was significantly higher than that of the FIB-4 index alone (AUC = 0.69, *Z* = 3.65, *p* < 0.01) and the APACHE II alone (AUC = 0.73, *Z* = 3.84, *p* < 0.01).

**Conclusion:**

This study confirms the independent prognostic value of the FIB-4 index, and its integration into a multivariable model provides a practical tool to improve early mortality risk stratification in acute pancreatitis patients.

## 1 Introduction

Acute pancreatitis represents a significant global health concern, with reported annual incidence rates ranging from 13 to 45 per 100,000 individuals (1–3). In the United States alone, it leads to approximately 275,000 hospital admissions each year, accompanied by an overall mortality rate of 2–8% (4). Outcomes of acute pancreatitis are closely associated with severity and the development of organ dysfunction, where mortality rises substantially in more severe cases (5, 6). Comorbidities further influence clinical trajectories and have been consistently linked to prognosis in acute pancreatitis (7, 8).

Chronic liver disease is highly prevalent and recognized as an important determinant of adverse outcomes in acute pancreatitis (9). In particular, liver cirrhosis has been shown to exacerbate severity in alcohol-induced acute pancreatitis, with a higher frequency observed in moderately severe and severe forms of the acute pancreatitis (10). Evidence indicates that concomitant cirrhosis markedly elevates the risk of infections, periprocedural complications, multiorgan failure, and mortality in acute pancreatitis patients (11). A study further reported that acute pancreatitis patients with coexisting cirrhosis experience significantly worse clinical outcomes, including venous thromboembolism, acute renal failure, shock, increased mortality, prolonged hospital stay, and higher total hospitalization costs compared to those without cirrhosis (12).

Liver fibrosis, as the essential precursor and pathological foundation of cirrhosis, remains insufficiently investigated in acute pancreatitis populations. The impact of liver fibrosis severity on prognosis in acute pancreatitis warrants further detailed analysis. The Fibrosis-4 index (FIB-4), a non-invasive marker of liver fibrosis, has been validated as a reliable indicator of fibrosis severity and has demonstrated prognostic relevance across various conditions, including heart failure, diabetes, Coronavirus Disease 2019 (COVID-19), acute coronary syndrome, and cholangiocarcinoma (13–20). This study aims to evaluate the prognostic utility of FIB-4 in patients with acute pancreatitis.

## 2 Materials and methods

### 2.1 Participants

This retrospective cohort study was approved by the Ethics Committee of West China Hospital, Sichuan University (Ethics Review Number: 2021-1694) and was conducted in accordance with the Declaration of Helsinki. The study population consisted of adult patients with acute pancreatitis admitted to the intensive care unit (ICU) of West China hospital between December 2016 and December 2019. This study adopted the diagnostic criteria recommended in the Atlanta classification (2012), requiring at least two of the three standard criteria (characteristic abdominal pain, serum amylase/lipase >3 times the upper limit of normal, or characteristic findings on cross-sectional imaging). Patients were admitted to our ICU primarily for: (a) new or worsening organ failure, (b) need for respiratory support (invasive or non-invasive), (c) requirement for high-dose vasoactive drugs, or (d) progressive clinical deterioration meeting SIRS criteria necessitating close monitoring. Patients were excluded if they had incomplete medical records, specifically missing laboratory data on liver enzymes or undocumented Acute Physiology And Chronic Health Evaluation II (APACHE II) scores (*n* = 12). After applying these criteria, 467 patients were included in the final analysis. Written informed consent for observational research participation was obtained from all subjects or their legally authorized representatives during the hospitalization process.

### 2.2 Data collection

All clinical and laboratory data for this retrospective study were extracted from the centralized electronic medical record repository of West China Hospital, Sichuan University. Patient baseline characteristics (age and sex) and documented comorbidities (hypertension and diabetes mellitus) were retrieved from admission registry records. All laboratory test results were derived from standardized venous blood specimens collected within the 6-h window following ICU admission; the APACHE II score was calculated based on data obtained during this same 6-h window. The FIB-4 score was computed using the validated formula: [Age (years) × AST (IU/L)]/[Platelet count (×10^9^/L) × ALT (IU/L)]. All patients included in the study completed the follow-up period. For patients who were discharged or transferred from the ICU within the 28-day observational window, vital status at the 28-day time point was ascertained through structured telephone interviews. The primary endpoint was defined as 28-day all-cause mortality following index hospitalization.

### 2.3 Statistical analysis

This study assessed data distribution using the Kolmogorov–Smirnov test. Categorical variables are summarized as frequencies with percentages, with intergroup comparisons performed using the Chi-square test or Fisher’s exact test as appropriate. Continuous variables are expressed as mean ± standard deviation when normally distributed and as median (interquartile range) when non-normally distributed; group differences were evaluated with the Student’s *t*-test and the Mann–Whitney *U* test, respectively. To identify independent predictors of 28-day mortality in acute pancreatitis, significant factors from the univariate analyses were subsequently entered into a multivariate logistic regression model. Variables retaining significance in the multivariate model were integrated into a logistic regression-based predictive model. The analysis was conducted using the rms (Regression Modeling Strategies) package in R software. The resulting model was visually presented as a nomogram, a graphical prediction model designed to facilitate its application in clinical practice. Receiver operating characteristic (ROC) curves were generated for both the FIB-4 index and the newly developed predictive model, and their respective areas under the curve (AUC) were computed. Pairwise comparisons of AUCs were performed using the DeLong test for correlated ROC curves.

A two-tailed *p*-value below 0.05 was defined as statistically significant. All statistical analyses and graphical presentations were conducted using SPSS version 22.0 (IBM Corp., Armonk, NY, USA) and R software (version 3.6.1; R Foundation for Statistical Computing, Vienna, Austria).

## 3 Results

### 3.1 Baseline information of enrolled acute pancreatitis patients

The analysis of baseline characteristics in the cohort of 467 acute pancreatitis patients showed non-survivors were older (*p* < 0.01) and had higher APACHE II scores at admission (*p* < 0.01) than survivors (Table 1). Non-survivors exhibited elevated laboratory parameters white blood cell (WBC) (*p* < 0.01), total bilirubin (*p* < 0.01), ALT (*p* < 0.01), AST (*p* < 0.01), amylase (*p* = 0.02), serum creatinine (*p* < 0.01), sodium (*p* = 0.03), potassium (*p* = 0.02), PT (prothrombin time) (*p* < 0.01) whereas reductions were observed in platelets (*p* < 0.01), albumin (*p* = 0.01), and calcium (*p* = 0.03). FIB-4 index was significantly elevated among non-survivors (*p* < 0.01). Both length of ICU stay and length of hospital stay were shorter among non-survivors (*p* < 0.01).

**Table 1:** Baseline of enrolled acute pancreatitis patients.

Variables	All patients (*n* = 467)	Survivors (*n* = 367, 78.6%)	Non-survivors (*n* = 100, 21.4%)	*p*-value
Age (years)	48.00 (37.00–56.00)	47.00 (36.00–54.00)	51.0 (44.00–66.00)	<0.01
Male (*n*, %)	304 (65.1%)	244 (66.5%)	60 (60.0%)	0.23
Hypertension (*n*, %)	82 (17.6%)	62 (16.9%)	20 (20.0%)	0.47
Diabetes mellitus (*n*, %)	85 (18.2%)	67 (18.3%)	18 (18.0%)	0.95
APACHE II	19 (13–25)	18 (13–22)	25 (19–30)	<0.01
WBC (10^9^/L)	11.94 (8.45–16.75)	11.69 (8.13–16.25)	13.39 (10.47–18.87)	<0.01
Platelet (10^9^/L)	161 (112–231)	170 (118–239)	134 (95–198)	<0.01
Hemoglobin (g/L)	113 (87–142)	114 (89–140)	107 (84–150)	0.70
Albumin (g/L)	32.90 (29.00–37.80)	33.40 (29.20–38.30)	31.60 (28.30–35.50)	0.01
Triglyceride (mmol/L)	2.66 (1.46–5.28)	2.69 (1.46–5.28)	2.49 (1.47–5.23)	0.53
Cholesterol (mmol/L)	3.38 (2.46–4.77)	3.45 (2.55–4.79)	3.22 (1.95–4.58)	0.05
Total bilirubin (umol/L)	17.80 (11.80–29.60)	16.80 (11.00–28.10)	22.60 (15.20–34.00)	<0.01
ALT (U/L)	27 (15–54)	25 (15–49)	38 (18–78)	<0.01
AST (U/L)	44 (25–82)	40 (24–74)	66 (38–122)	<0.01
Lipase (U/L)	207 (56–1,018)	197 (60–893)	446 (54–1,317)	0.24
Amylase (U/L)	203 (56–909)	182 (55–784)	371 (70–1,063)	0.02
Serum creatinine (umol/L)	89 (57–185)	78 (55–154)	133 (79–281)	<0.01
Sodium (mmol/L)	138.60 (134.40–142.70)	138.10 (134.30–142.20)	139.50 (135.60–144.20)	0.03
Potassium (mmol/L)	4.01 (3.55–4.50)	3.99 (3.54–4.44)	4.16 (3.72–4.72)	0.02
Chloride (mmol/L)	105.80 (100.80–110.20)	105.70 (100.90–110.00)	106.30 (100.60–110.40)	0.64
Calcium (mmol/L)	1.93 (1.75–2.12)	1.96 (1.78–2.12)	1.86 (1.66–2.08)	0.03
PT (s)	13.80 (12.70–15.40)	13.70 (12.70–15.10)	14.70 (13.10–16.30)	<0.01
FIB-4 index	2.65 (1.27–5.03)	2.28 (1.16–4.43)	4.05 (2.48–8.29)	<0.01
Length of ICU stay (day)	14 (7–27)	15 (8–30)	12 (5–18)	<0.01
Length of hospital stay (day)	23 (14–38)	28 (17–42)	15 (8–22)	<0.01

APACHE II, Acute Physiology and Chronic Health Evaluation II; WBC, white blood cell; ALT, alanine aminotransferase; AST, aspartate aminotransferase; PT, prothrombin time; FIB-4, Fibrosis-4.

### 3.2 Logistic regression of risk factors for the 28-day mortality of acute pancreatitis patients

The results of the logistic regression analysis were presented as Table 2. In the univariate analysis, age (*p* < 0.01), APACHE II score (*p* < 0.01), WBC (*p* < 0.01), platelet count (*p* < 0.01), albumin (*p* < 0.01), total bilirubin (*p* = 0.04), ALT (*p* = 0.01), AST (*p* = 0.01), serum creatinine (*p* < 0.01), sodium (*p* < 0.01), potassium (*p* = 0.01), prothrombin time (*p* < 0.01), and the FIB-4 index (*p* < 0.01) were significantly associated with 28-day mortality. In the multivariate analysis, after adjusting for potential confounders, age (*p* < 0.01), APACHE II (*p* < 0.01), WBC (*p* = 0.02), sodium (*p* = 0.03), and the FIB-4 index (*p* = 0.03) remained independent predictors of 28-day mortality.

**Table 2:** Logistic regression analysis of risk factors for the 28-day mortality of acute pancreatitis patients.

Variables	Univariate logistic regression			Multivariate logistic regression		
	OR	95% CI	*p*-value	OR	95% CI	*p*-value
Age	1.03	1.02–1.05	<0.01	1.03	1.01–1.05	<0.01
Male	0.76	0.48–1.19	0.23			
Hypertension	1.23	0.70–2.16	0.47			
Diabetes mellitus	0.98	0.55–1.75	0.95			
APACHE II	1.12	1.08–1.15	<0.01	1.10	1.06–1.14	<0.01
WBC	1.05	1.01–1.08	<0.01	1.05	1.01–1.09	0.02
Platelet	1.00	0.99–1.00	<0.01	1.00	1.00–1.00	0.26
Hemoglobin	1.00	0.99–1.01	1.00			
Albumin	0.96	0.93–0.99	0.01	0.99	0.95–1.01	0.52
Triglyceride	0.98	0.96–1.01	0.27			
Total cholesterol	0.98	0.98–1.04	0.53			
Total bilirubin	1.01	1.00–1.01	0.04	1.00	1.00–1.01	0.52
ALT	1.00	1.00–1.00	0.01	1.00	0.99–1.00	0.71
AST	1.00	1.00–1.00	0.01	1.00	1.00–1.00	0.65
Lipase	1.00	1.00–1.00	0.76			
Amylase	1.00	1.00–1.00	0.84			
Serum creatinine	1.00	1.00–1.00	<0.01	1.00	1.00–1.00	0.95
Sodium	1.04	1.01–1.07	<0.01	1.04	1.01–1.08	0.03
Potassium	1.38	1.07–1.79	0.01	1.28	0.90–1.79	0.17
Chloride	1.00	0.99–1.01	0.72			
Calcium	0.54	0.28–1.07	0.08			
PT (s)	1.10	1.04–1.17	<0.01	1.06	1.01–1.14	0.07
FIB-4 index	1.13	1.08–1.19	<0.01	1.09	1.01–1.19	0.03

OR, odds ratio; CI, confidence interval; APACHE II, Acute Physiology and Chronic Health Evaluation II; WBC, white blood cell; ALT, alanine aminotransferase; AST, aspartate aminotransferase; PT, prothrombin time; FIB-4, Fibrosis-4.

### 3.3 FIB-4 and predictive model for the 28-day mortality of acute pancreatitis patients

**Figure 1:**
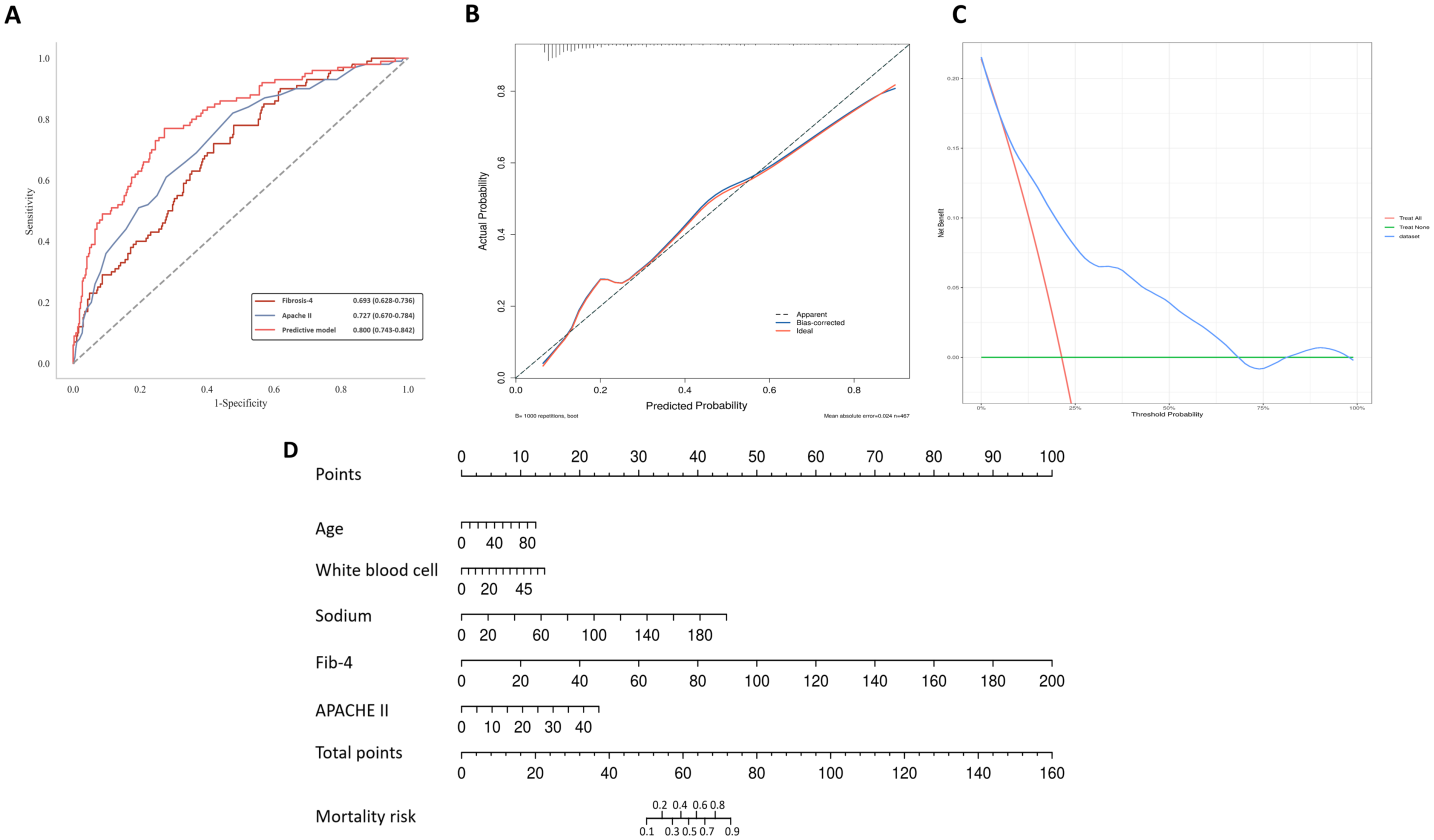
Development and comprehensive evaluation of the predictive model for mortality risk. **(A)** Receiver Operating Characteristic (ROC) curves demonstrating the discrimination performance of the predictive model compared to the APACHE II and FIB-4 models. **(B)** Calibration plot showing good agreement between the predicted and actual mortality probabilities. **(C)** Decision curve analysis (DCA) illustrating the net benefit of the predictive model across different threshold probabilities. **(D)** Clinical nomogram incorporating variables such as Age, White blood cell count, Sodium, FIB-4, and APACHE II to calculate total points and estimate mortality risk.

The final predictive model for acute pancreatitis 28-day mortality was constructed by integrating five independent factors identified in multivariate logistic regression: age, WBC, sodium level, FIB-4 index, and APACHE II score (Table 3). Collinearity diagnostics showed no substantial multicollinearity among the predictors included in the multivariable model, with all tolerance values >0.9 and all VIF values close to 1 (Supplementary Table S1). This combined model demonstrated an AUC of 0.80 (0.74–0.84), which was significantly higher than that of the FIB-4 index alone (AUC = 0.69; *Z* = 3.65, *p* < 0.01) and the APACHE II alone (AUC = 0.73; *Z* = 3.8, *p* < 0.01) (Table 4, Supplementary Tables S2, S3, Figure 1A). No statistically significant difference in discriminative ability was observed between the FIB-4 index and APACHE II (*Z* = 0.90, *p* = 0.37). The Brier score of the predictive model was 0.1305, indicating an overall low prediction error and acceptable agreement between predicted and observed outcomes (Figure 1B). In addition, decision curve analysis demonstrated that the prediction model achieved a higher net benefit than the treat-all and treat-none strategies over a broad range of threshold probabilities, particularly between approximately 5 and 65% (Figure 1C). A clinical nomogram incorporating these five predictors was developed for practical risk estimation (Figure 1D) and the final logistic regression equation is as follows: Logit(P) = −11.862 + 0.096 × FIB-4 + 0.045 × WBC + 0.043 × sodium + 0.027 × age + 0.100 × APACHE II.

**Table 3:** Predictive model based on logistic regression for the 28-day mortality of acute pancreatitis patients.

Variables	OR	95% CI	*p*
Age	1.03	1.01–1.05	<0.01
WBC	1.05	1.01–1.08	<0.01
Sodium	1.04	1.01–1.08	0.01
FIB-4	1.10	1.05–1.16	<0.01
APACHE II	1.11	1.07–1.15	<0.01

OR, odds ratio; CI, confidence interval; APACHE II, Acute Physiology and Chronic Health Evaluation II; WBC, white blood cell; FIB-4, Fibrosis-4.

**Table 4:** AUC value of Fibrosis-4 and developed predictive model for the 28-day mortality of acute pancreatitis patients.

Variables	AUC	Sensitivity	Specificity	Best cut-off value
FIB-4	0.69 (0.63–0.74)	0.78	0.52	2.45
APACHE II	0.73 (0.67–0.78)	0.82	0.52	19.00
Predictive model	0.80 (0.74–0.84)	0.77	0.73	0.20

APACHE II, Acute Physiology and Chronic Health Evaluation II; FIB-4, Fibrosis-4.

## 4 Discussion

The present study identified several key clinical and laboratory parameters significantly associated with 28-day mortality in a cohort of 467 acute pancreatitis patients. Notably, our analysis revealed that the FIB-4 index, a readily calculable and non-invasive composite score, is an independent predictor of 28-day mortality in this population. The significant association between elevated FIB-4 and increased mortality suggests that the composite derangement of age, liver enzymes, and platelet count—whether stemming from underlying chronic liver disease, the acute hepatic and hematologic insult of severe pancreatitis, or both—identifies a high-risk phenotype not fully captured by traditional severity scores. The pathophysiological link between elevated FIB-4 and mortality likely involves multiple mechanisms. If FIB-4 elevation reflects underlying liver fibrosis, this indicates reduced hepatic reserve, which could compromise essential metabolic and detoxification functions during severe acute pancreatitis (21–23). Concurrently, the acute AST/ALT elevations and thrombocytopenia contributing to high FIB-4 scores directly mark severe systemic inflammation, coagulopathy, and hypoperfusion, key drivers of multiorgan failure and death. Furthermore, a state of hepatic dysfunction and hematologic disturbance, as inferred from a high FIB-4, is associated with complications such as hypoalbuminemia and coagulation factor deficiencies, which may elevate mortality by impairing nutritional status and predisposing to hemorrhagic complications (24–26). More broadly, an elevated FIB-4 likely signifies a systemic pro-inflammatory and pro-fibrotic milieu (encompassing both chronic liver disease and acute critical illness), which could exacerbate the dysregulated immune response and end-organ damage characteristic of severe acute pancreatitis (27–29).

In addition to the FIB-4, another four independent risk factors confirmed in this study were age, WBC, sodium, and APACHE II. Advanced age impairs organ reserve and immune function, predisposing elderly patients to systemic progression from localized inflammation to multi-organ failure, thereby elevating mortality and complications (30–32). Hypernatremia indicates dehydration and hyperosmolarity, reflecting hypovolemia and renal hypoperfusion that exacerbate pancreatic necrosis and systemic injury (33, 34). WBC signifies systemic inflammation, where excessive activation triggers a cytokine storm and organ dysfunction, while immune impairment increases infection risk (35, 36). Validated in multiple studies, WBC elevation reliably predicts outcomes of acute pancreatitis (37, 38). The APACHE II score objectively quantifies physiological derangement, correlating with inflammation severity and organ dysfunction risk to forecast mortality and complications (39, 40).

The constructed predictive model, incorporating the FIB-4 index into a framework with age, WBC, sodium, and APACHE II, achieved an AUC of 0.800, representing a statistically significant improvement over the APACHE II score alone (AUC = 0.727). This enhancement underscores the additive value of including a marker of chronic liver pathology. While the APACHE II is a comprehensive physiological score, it is primarily a snapshot of acute dysfunction. The FIB-4 index, in contrast, provides information on the patient’s pre-morbid hepatic health, a dimension of vulnerability that complements the assessment of acute physiological stress. The comparable AUCs of FIB-4 (0.693) and APACHE II (0.727) in isolation are noteworthy, suggesting that this simple, non-invasive, and cost-free calculation holds meaningful prognostic information that approximates a complex, multi-parameter ICU score in this specific context.

The clinical utility of our findings is operationalized through the developed nomogram, which allows for bedside estimation of individual mortality risk by summing points assigned to each of the five predictors. This tool could aid clinicians in early identification of high-risk acute pancreatitis patients who might benefit from more intensive monitoring, aggressive supportive care, or timely interventions.

Several limitations of this study must be acknowledged. First, this study has a retrospective, single-center design, and its findings require validation in prospective studies across different populations and healthcare centers. Prospective, multicenter external validation is necessary before considering its application in clinical practice. Second, as the study exclusively enrolled patients admitted to the ICU, the cohort represents a critically ill subpopulation of acute pancreatitis. While we have provided the clinical indications for ICU admission, the inherent selection bias limits the generalizability of our findings to all acute pancreatitis patients, particularly those with mild or moderately severe disease. Therefore, the results and conclusions should be interpreted within the context of a critically ill population. Third, a major limitation relates to interpreting FIB-4 in acute critical illness. Although validated as a liver fibrosis marker in chronic liver disease, elevated FIB-4 in severe acute pancreatitis likely reflects both chronic liver burden and acute insults—such as sepsis, ischemic hepatitis, or coagulopathy—that alter its components (AST, ALT, platelets). Thus, while prognostic, we cannot determine how much of its predictive value stems from fibrosis versus acute injury. Future studies using specific chronic liver disease markers (e.g., elastography) are needed to clarify this distinction and assess whether modifying the high-risk state indicated by FIB-4 improves outcomes.

## 5 Conclusion

In this study, the FIB-4 index demonstrated an independent association with 28-day mortality in critically ill patients with acute pancreatitis admitted to the ICU, and showed potential to enhance early risk stratification when added to established prognostic factors. However, these findings are considered exploratory and require prospective, multicenter validation to assess their clinical utility and impact on patient outcomes.

## Data Availability

The raw data supporting the conclusions of this article will be made available by the authors, without undue reservation.
