# Supplementary material for: Association between the Fibrosis-4 index and mortality risk in acute pancreatitis
Source: Front Med (Lausanne). 2026 May 8;13:1767483. doi: 10.3389/fmed.2026.1767483 (PMC13194500; doi:10.3389/fmed.2026.1767483)
Supplement: Supplementary file 1 [file Table_1.DOCX]

| **Table S1. Multicollinearity diagnostics of predictors included in the multivariable logistic regression model** | | |
| --- | --- | --- |
| Variables | Tolerance | VIF |
| Age | 0.98 | 1.02 |
| WBC | 0.98 | 1.02 |
| Sodium | 0.99 | 1.01 |
| FIB-4 index | 0.98 | 1.02 |
| APACHE II | 0.97 | 1.03 |
| VIF, Variance Inflation Factor; APACHE II, Acute Physiology And Chronic Health Evaluation II; WBC, White blood cell; FIB-4, Fibrosis-4. | | |

| **Table S2. Z value of AUC comparison using Z test** | | | |
| --- | --- | --- | --- |
|  | FIB-4 | APACHE II | Predictive model |
| FIB-4 |  | 0.902 | 3.650 |
| APACHE II | 0.902 |  | 3.842 |
| Predictive model | 3.650 | 3.842 |  |
| FIB-4, Fibrosis-4; APACHE II, Acute Physiology And Chronic Health Evaluation II. | | | |

| **Table S3. P value of AUC comparison using Z test** | | | |
| --- | --- | --- | --- |
|  | FIB-4 | APACHE II | Predictive model |
| FIB-4 |  | 0.367 | <0.001 |
| APACHE II | 0.367 |  | <0.001 |
| Predictive model | <0.001 | <0.001 |  |
| FIB-4, Fibrosis-4; APACHE II, Acute Physiology And Chronic Health Evaluation II. | | | |
